# Fractionated stereotactic conformal radiotherapy for large benign skull base meningiomas

**DOI:** 10.1186/1748-717X-6-36

**Published:** 2011-04-12

**Authors:** Giuseppe Minniti, Enrico Clarke, Luigi Cavallo, Mattia Falchetto Osti, Vincenzo Esposito, Gianpaolo Cantore, Paolo Cappabianca, Riccardo Maurizi Enrici

**Affiliations:** 1 Department of Radiation Oncology, Sant' Andrea Hospital, Università degli Studi di Roma "La Sapienza", Rome, Italy; 2 Department of Neurological Sciences, Division of Neurosurgery, Università degli Studi di Napoli Federico II, Naples, Italy; 3 Department of Neurosurgical Sciences, Division of Neurosurgery, Neuromed Institute, Pozzilli (IS), Italy

## Abstract

**Purpose:**

to assess the safety and efficacy of fractionated stereotactic radiotherapy (FSRT) for large skull base meningiomas.

**Methods and Materials:**

Fifty-two patients with large skull base meningiomas aged 34-74 years (median age 56 years) were treated with FSRT between June 2004 and August 2009. All patients received FSRT for residual or progressive meningiomas more than 4 centimeters in greatest dimension. The median GTV was 35.4 cm^3 ^(range 24.1-94.9 cm^3^), and the median PTV was 47.6 cm^3 ^(range 33.5-142.7 cm^3^). Treatment volumes were achieved with 5-8 noncoplanar beams shaped using a micromultileaf collimator (MLC). Treatment was delivered in 30 daily fractions over 6 weeks to a total dose of 50 Gy using 6 MV photons. Outcome was assessed prospectively.

**Results:**

At a median follow-up of 42 months (range 9-72 months) the 3-year and 5-year progression-free survival (PFS) rates were 96% and 93%, respectively, and survival was 100%. Three patients required further debulking surgery for progressive disease. Hypopituitarism was the most commonly reported late complication, with a new hormone pituitary deficit occurring in 10 (19%) of patients. Clinically significant late neurological toxicity was observed in 3 (5.5%) patients consisting of worsening of pre-existing cranial deficits.

**Conclusion:**

FSRT as a high-precision technique of localized RT is suitable for the treatment of large skull base meningiomas. The local control is comparable to that reported following conventional external beam RT. Longer follow-up is required to assess long term efficacy and toxicity, particularly in terms of potential reduction of treatment-related late toxicity.

## Introduction

The optimal management of large benign meningiomas of the skull base is challenging. Surgery remains the standard treatment and following apparently complete removal the reported control rates are in the region of 95% at 5 years and 90% at 10 years [1-14]. However, a significant subset of meningiomas, especially large tumors involving the cavernous sinus, the petroclival region, and the brainstem cannot be completely resected for the risk of significant morbidity [1,3-6,9]. In such patients incomplete removal of tumor with preservation of the involved cranial nerves may result in improved neurological function and temporary local control, although progression on long-term follow-up is reported in up to 80% of patients [2,8,13].

Local control following partial resection of benign meningiomas and at the time of recurrence can be improved with conventional fractionated external beam radiotherapy (RT), with a reported 10-year progression-free survival in the region of 75-90% [15-17]. More recently, stereotactic radiation techniques in form of stereotactic radiosurgery (SRS) and fractionated stereotactic radiotherapy (FSRT) have been developed as accurate techniques that can deliver more localized irradiation with a steeper dose gradient between the tumor and the surrounding normal tissue, and consequently reducing the volume of normal brain irradiated to high radiation doses. Both techniques have been reported as an effective treatment in several benign skull base tumors including pituitary adenomas [18,19], acoustic neuromas [20,21], craniopharyngiomas [22,23] and meningiomas [24].

Although in many centers SRS is the preferred treatment option for patients with small to moderate recurrent or enlarging skull base meningiomas, fractionated RT is often performed for larger tumors close to critical structures because of the radiobiological advantage of dose fractionation in reducing the risk of post-radiation long-term complications. In this study, we report the experience with FSRT at our center for patients with large residual or progressive skull base meningiomas.

## Patients and Methods

Between June 2004 and August 2009, fifty-two patients with benign skull base meningiomas more than 4 centimeters in greatest dimension were treated with FSRT. Characteristics of patients are shown in Table 1. There were 17 males and 35 females. Median age was 56 years, ranging from 34 to 74 years. All patients had a KPS ≥ 60. Eighteen received FSRT following surgery for a macroscopic tumor remnant and the other 34 patients had FSRT at tumor regrowth. Twenty-one patients had more than one surgical procedure. Histology confirmed the presence of a benign meningioma in all cases. Cranial nerve deficits were present in 43 patients, mainly consisting of visual field defects in 22 and/or impaired ocular motility in 19 (Table 2). Endocrine deficits were present in 7 patients. The median time from resection to FSRT was 39 months. None of patients had received previous radiotherapy.

**Table 1:** Clinical characteristics of 52 patients with large skull base meningiomas treated with fractionated stereotactic radiotherapy

**Sex**	17 M\35 F
**Median age (range)**	56 yrs (34-74)
**KPS**	
70-80	
90-100	
**Neurological deficits**	43
**Number of surgeries**	
1	31
2	16
3	5
**Pituitary function**	
normal	45
hypopituitarism	7
**Tumor site**	
cavernous sinus	38
petroclival	6
Sphenoid wing	4
cerebellopontine angle	5
**Gross tumour volume (GTV)**	
Median	35.4 cm^3^
Range	24.1 - 94.9 cm^3^
**Planning target volume (PTV)**	
Median	47.6 cm^3^
Range	33.5 -142.7 cm^3^

MRI, magnetic resonance images;

**Table 2:** Cranial deficits at presentation in 52 patients with large skull base benign meningiomas

Cranial deficits*	Before FSRT	After FSRT (median follow-up 34 months)
II	22	16**
III	10	8
IV	2	2
V	9	6
VI	11	8
VII	5	3
VIII	4	6**

*14 patients had multiple cranial deficits at presentation

** One patient with visual field defect and 2 patients with hearing loss had clinical deterioration during the follow-up.

### SCRT technique and dose prescription

The FSRT technical details and procedure using the BrainLab stereotactic mask fixation system have been previously reported [25]. The gross tumor volume (GTV) was delineated on the basis of the contrast-enhancing tumor demonstrated on T1-weighted MRI fused with the simulation CT images. CTV was considered the same as GTV. The planning target volume (PTV) was generated by the geometric expansion of GTV plus 2-3 mm. For the last 18 patients the 3-D margin was reduced to 2 mm. The median GTV was 35.4 cm^3 ^(range 24.1-94.9 cm^3^). The PTV was 47.6 cm^3 ^(range 33.5-142.7 cm^3^). Treatment volumes were achieved with 5-8 noncoplanar beams shaped using a micromultileaf collimator (MLC). Plans were prescribed at the isocentre according to ICRU 50 criteria with PTV covered by the 95% isodose in 3-D. To assess the accuracy of relocation, the isocentre position was verified with a second CT scan performed just prior to the start of treatment. The tolerance of relocation had to be < 1.5 mm in any direction. Daily portal images acquired at 0 and 90° through the isocenter were obtained for each patient during the treatment. All patients were treated on a 6-MV LINAC with a 120 leaf MLC (Varian Clinac 600 DBX) and received a dose of 50 Gy in 30 fractions over 6 weeks.

### Follow-up and data analysis

A clinical assessment of neurological status and tolerance to treatment was performed every six months. An MRI scan was performed every 6 months for the first 2 years and thereafter every 12 months. Tumor control was defined by the absence of radiological tumor progression. Pituitary function was assessed by complete basal hormonal assessment and dynamic testing, as appropriate, in an endocrine clinic. Vision was assessed by serial ophthalmologic examinations. Tumor control and overall survival were measured from the start of FSRT. Univariate analysis and multivariate Cox proportional hazards regression model were used to test the effect of prognostic factors on tumor control.

## Results

### Tumor control and survival

**Figure 1:**
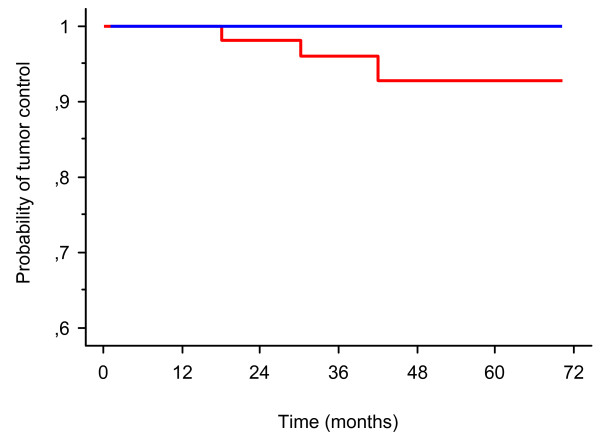
**Kaplan-Meier analysis of tumor control (red line) and overall survival (blue line) rates after FSRT of 52 large benign skull base meningiomas**.

**Figure 2:**
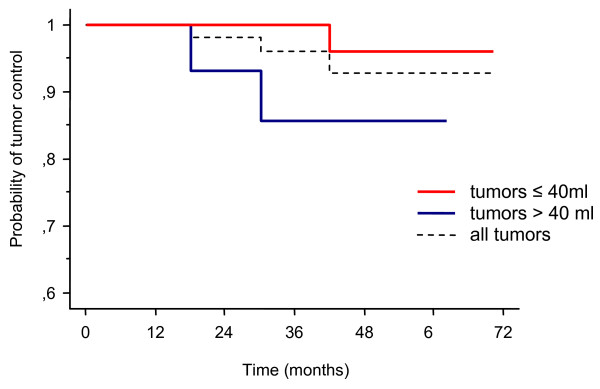
**Kaplan-Meier analysis of tumor control in patients with large benign skull base meningiomas treated with fractionated stereotactic radiotherapy (FSRT) according to tumor volume (≤ 40 ml *versus *> 40 ml) (p = 0.16)**.

Fifty-two patients with large benign skull base meningioma were treated with FSRT. At the time of analysis in December 2010, three patients had tumor progression 18, 30 and 42 months after FSRT and required surgery. After a median follow-up of 42 months (range from 12 months to 72 months), the actuarial tumor control was 96% at 3 years and 93% at 5 years, and respective survival 100% (Figure 1). Local control was similar for patients treated with FSRT as a part of their primary treatment or at the time of recurrence. MRI showed on serial imaging no changes in 37 (71%) and reduction in tumor size in 12 patients (23%), however without reaching conventional partial response criteria. *Univariate analysis showed no significant tumor control between 23 meningiomas larger than 40 cm^3 ^and 29 meningiomas smaller than or equal to 40 cm^3 ^(P = 0.16) *(Figure 2**)**. Similarly, tumor location, sex, and age were not correlated with tumor control.

### Neurological function

Neurological deficits were present in 43 (82%) patients. After FSRT 11 (20%) patients had a clinical improvement of neurological deficits (Table 2). Vision improved in 7 patients and cranial nerve function in 5 patients. The optic chiasm was included in the PTV of the majority of patients (n = 36) and received the prescribed dose of 50 Gy. Three patients deteriorated without evidence of tumor progression on imaging. One patient had a slight worsening of vision and two progressive hearing loss. Seven patients had a transient mild visual deterioration (n = 4) and a worsening of pre-existing 7^th ^(n = 1) and 5^th ^(n = 2) nerve palsy during or shortly after treatment, with full recovery after a short course of corticosteroids.

### Acute and late toxicity

All patients noted transient localized alopecia at the beam entrance with full subsequent recovery of hair growth. Tiredness occurred in 14 (27%) patients, lasting for 4-8 weeks after FSRT. Transient headache occurred in 6 patients. One patient had an increase in seizure frequency. A development of new or worsening of pre-existing hypopituitarism occurred in 10 (19%) patients after a median follow-up of 36 months, requiring hormone replacement therapy with gonadal steroids and growth hormone in 7 patients, GH replacement in 2 patients, and thyroxine and hydrocortisone in 4 patients. The pituitary fossa contained residual tumor in 27 patients, and was included in the PTV. New clinically apparent neurocognitive dysfunction (Grade II RTOG memory impairment) was reported in one patient. No radiation necrosis, cerebrovascular accidents and second tumors were reported.

## Discussion

Fifty-two patients with large skull base meningiomas were treated with FSRT between June 2004 and August 2009 at University of Rome "La Sapienza". At a median follow-up of 42 months tumor control rates were 96% and 93% at 3 and 5 years, with respective survival of 100%. The rate of complications was acceptable, consisting mainly in worsening vision in 2 patients and pituitary hormone deficits in 10 patients. *The reported tumor control rate is similar to that shown recently by others following conventional RT *[15-17,26-33] or FSRT [34-45](Table 3)*, however longer follow-ups needed to confirm the excellent results obtained in our series*. Of note, in our study we have considered only patients presenting with large benign meningiomas more than 4 centimeters in greatest dimension, with a median volume of 35.4 cm^3^. Milker-Zabel et al. [39] reported on 317 patients with benign skull base meningiomas of a median volume of 34 cm^3 ^treated with FSRT at University of Heidelberg. At a median follow-up of 5.7 years, 5-year and 10-year tumor control rates were 90.5% and 89%, and respective survival were 95% and 90%. Hamm et al [42] reported on 183 patients with large skull base meningiomas up to 135 cm^3 ^or close to optical structures treated with FSRT with a median dose of 56 Gy with daily fractions of 1.8-2 Gy. At a median follow-up of 36 months the overall survival and the progression-free survival rates for 5 years were 92.9%, and 96.9%, respectively. Grade II-III late neurological toxicity occurred in 5.5% of patients. A similar tumor control has been reported in patients with skull base meningiomas treated with conventional RT, however with an increased risk of recurrence for larger tumors [15,28,29]. Connell et al [29] reported a 5-year control of 93% for 54 patients with skull base meningiomas less than 5 centimeters in greatest dimension and 40% for tumors more than 5 centimeters, and similar findings have been reported by others [15,28]. *Although a clear limitation of the study is represented by the relatively short follow-up, neverthless our results and data from literature suggest that FSRT is an appropriate treatment option for patients with large recurrent or enlarging skull base meningiomas with a 5-year control similar or even better than conventional RT*.

**Table 3:** Summary of main results on published studies on the conformal radiotherapy and FSRT of skull base meningiomas

authors	Patients (n)	Technique (%)	Volume (ml)	Dose (Gy)	Follow-up (months)	Local control (%)	Late toxicity (%)
Goldsmith et al., 1994	117	CRT	NA	54	40	89 at 5 and 77 at 10 years	3.6
Maire et al., 1995	91	CRT	NA	52	40	94	6.5
Peele et al., 1996	42	CRT	NA	55	48	100	5
Condra et al.,1997	28	CRT	NA	53.3	98	87 at 15 years	24
Connell et al., 1999	54	CRT	NA	54	55	76 at 5 years	19
Maguire et al., 1999	26	CRT	NA	53	41	8 at 8 years	8
Nutting et al., 1999	82	CRT	NA	55-60	41	92 at 5 and 83 at 10 years	14
Vendrely et al., 1999	156	CRT	NA	50	40	79 at 5 years	11.5
Dufour et al., 2001	31	CRT	NA	52	73	93 at 5 and 10 years	3.2
Pourel et al., 2001	28	CRT	NA	56	30	95 at 5 years	4
Mendenhall et al., 2003	101	CRT	NA	54	64	95 at 5, 92 at 10 and 15 years	8
Debus et al., 2001	189	FSRT	52.5	56.8	35	97.3	1.6
Jalali et al., 2002	41*	FSRT	17.9	55	21	100	12.1
Lo et al., 2002	18*	FSRT	8.8	54	30.5	93.3	5
Torres et al., 2003	77*	FSRT	16.1	48.4	24	97.2	5.2
Selch et al., 2004	45	FSRT	14.5	56	36	100 at 3 years	0
Milker-Zabel et al., 2005	317*	FSRT	33.6	57.6	67	90.5 at 5 and 89 at 10 years	8.2
Henzel et al., 2006	84	FSRT	11.1	56	30	100	NA
Brell et al., 2006	30	FSRT	11.3	52	50	93 at 4 years	6.6
Hamm et al., 2008	183	FSRT	27.4	56	36	97 at 5 years	8.2
Litré et al., 2009	100	FSRT	NA	45	33	94 at 3 years	0
Metellus et al., 2010	47	FSRT	12.6	52.9	82.8	98 at 5 and 96 at 10 years	2.6
Tanzler E et al., 2010	144**	FSRT	NA	52.7	96	97 at 5 and 96 at 10 years	7

CRT, conformal 3-D radiotherapy; FSRT, fractionated stereotactic radiotherapy

*Series include some patients with intracranial meningiomas

** 41 patients received CRT

The external beam radiation dose for meningioma that represents the best balance of tumor control and a low complication rate has not been defined. Most of published series show no significant difference on tumor control with the use of doses ranging between 50 and 60 Gy, however a dose < 50 Gy has been associated with higher recurrence rates [15,27,33]. The present results, with a tumor control of 90% at 5 years, suggest that a dose of 50 Gy in 30 fractions may achieve a good local tumor control with acceptable toxicity in large skull base meningiomas.

SRS represents an effective and safe alternative treatment option for patients with skull base meningiomas. At doses of 12-16 Gy the reported actuarial 5-year and 10-year tumor local control rates are in the range of 90-95% and 80-85%, as shown in some recent large series [46-58]; however, larger tumors are associated with worse long-term local control and increased toxicity [49,54,55]. DiBiase et al [49] reported a significant higher 5-year tumor control in patients with meningiomas < 10 ml than those with larger tumors (92% vs 68%, p = 0.038). In a large series of 972 patients with meningioma treated with Gamma Knife SRS using a median dose to the tumor margin of 13 Gy local control was negatively correlated with increasing volume (p = 0.01), and a similar trend was observed with disease-specific survival (p = 0.11) [54]. In a retrospective review of 116 patients treated with SRS for meningiomas > 10 cm^3 ^in volume at a dose of 15 Gy, Bledsoe et al [55] reported a local control of 92% at 7 years, although complications occurred in 18% of patients with skull base tumors. Interestingly, Iwai et al [53] using a median marginal dose ranging from 8 to 12 Gy showed a progression-free survival of 93% and 83% at 5 and 10 years in 108 patients with skull base meningiomas treated with Gamma Knife SRS; permanent neurological deficits occurred in 6% of patients. Although the use of radiosurgical doses less than 12 Gy may represent a promising approach in patients with large meningiomas, the reported favourable outcome needs to be confirmed in future studies. Currently, results from published series suggest that FSRT is a better treatment option in such patients based on its proven efficacy and safety.

Hypopituitarism was the most commonly reported late complication. A new pituitary hormone deficit requiring hormone replacement occurred in 19% of patients. Late neurological toxicity was observed in 7% of patients and consisted of worsening of pre-existing cranial deficits in 3 patients and mild neurocognitive dysfunction in one patient. A neurological improvement was observed in 19% of patients; vision remained stable in 46 patients and improved in 7 patients with visual impairment. Since the late effects of radiotherapy in terms of normal tissue damage expressed as radiation optic neuropathy occur usually within 1-5 years of treatment, the low incidence of radiation-induced optic neuropathy and others cranial nerve deficits at a median follow-up of 42 months provide some reassurance about the safety of the present dose and technique for large skull base meningiomas. The present and some other recent series on FSRT [34-45] and conformal RT [15,17] definitely contradict the historical perception of unresponsiveness of meningiomas as well as the considerably concern of high late morbidity following the radiation treatment for benign brain tumors, which was primarily based on old reports where radiation was delivered with orthovoltage machines.

We conclude that FSRT is a high precise and safe treatment for the majority of large skull base meningiomas, *with a control of tumor growth at 5 years comparable to that seen following conventional fractionated radiotherapy*. For patients with large skull base meningiomas a combination of conservative surgery and postoperative irradiation should always be considered when an attempt to complete resection carries unacceptable risks of neurological deficits. The use of 2-3 mm margin from GTV to generate PTV with FSRT permits a more localized irradiation compared with conventional radiotherapy. *Minimizing the radiation dose to normal brain FSRT may reduce the risk of developing late radiation-induced toxicity; however, the potential benefit in reducing long term treatment complications while maintaining a high efficacy will require longer follow-up of a large cohort of patients*.

## Competing interests

The authors declare that they have no competing interests.

## Authors' contributions

GM conceived of the study, participated in its design and coordination, and drafted the manuscript. LV and VE participated in study design, analysis and interpretation of data, and helped to draft the manuscript. EC and MFO performed the statistical analysis and participated in acquisition and analysis of data. PC, GC and RME critically reviewed/revised the article. All authors read and approved the final manuscript.
